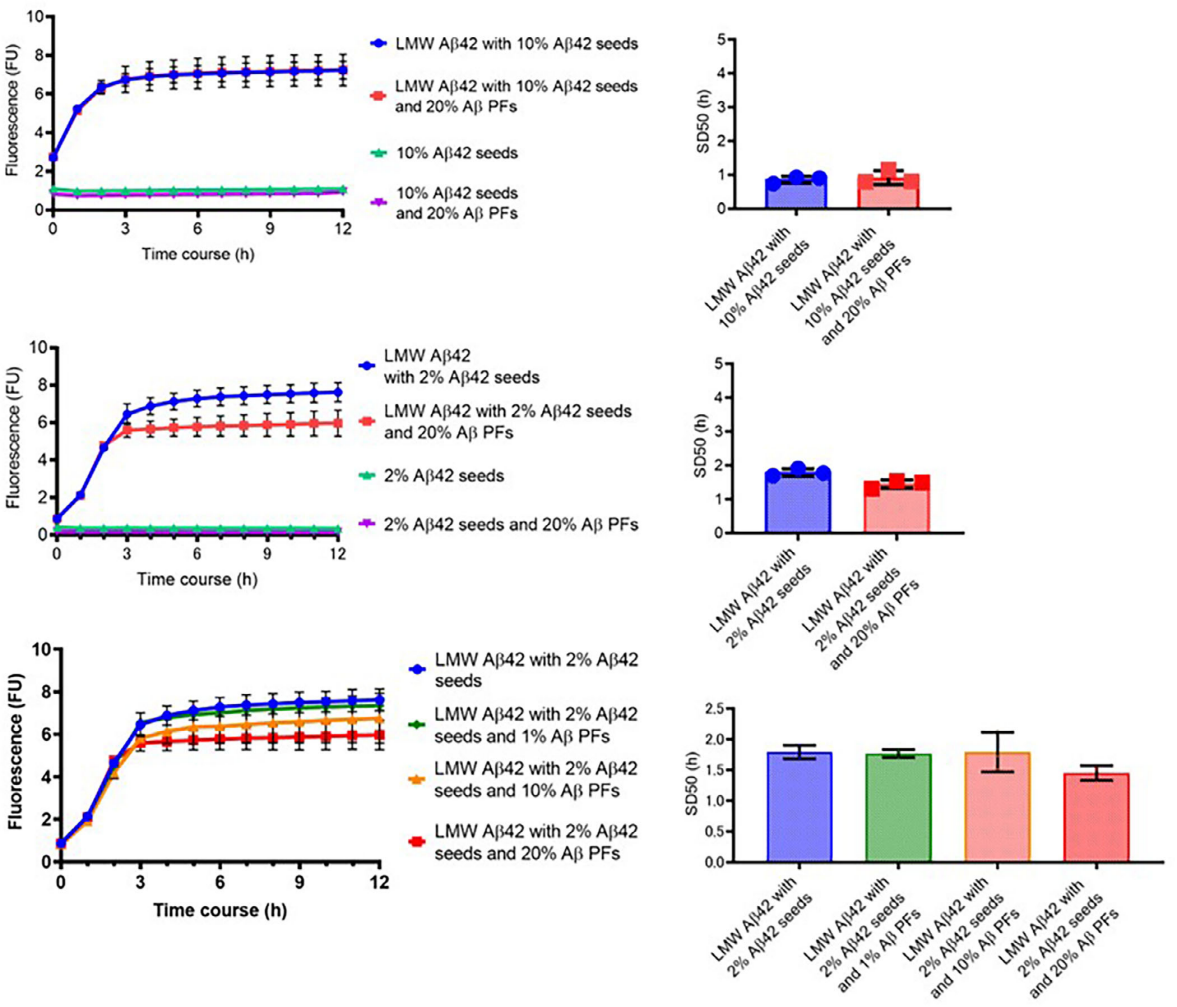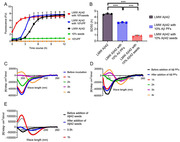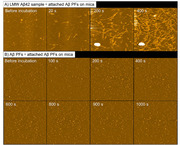# Possible Mechanisms of Amyloid β‐Protein Protofibrils for Acceleration of Aggregation

**DOI:** 10.1002/alz.085834

**Published:** 2025-01-03

**Authors:** Hiroto Nakano, Takahiro Nakayama, Sadao Hikishima, Makoto Mori, Jota Minamikawa, Daiki Muramatsu, Yasuhiro Sakashita, Tokuhei Ikeda, Moeko Shinohara, Kenjiro Ono

**Affiliations:** ^1^ Department of Neurology, Kanazawa University Graduate School of Medical Sciences, Kanazawa Japan; ^2^ World Premier International Research Center Initiative (WPI)‐Nano Life Science Institute, Kanazawa Japan; ^3^ Department of Neurology, Kanazawa University Graduate School of Medical Sciences, Kanazawa, Ishikawa Japan; ^4^ Kanazawa University Graduate School of Medical Sciences, Kanazawa Japan

## Abstract

**Background:**

The growing number of AD patients is a public concern all over the world. During the decade, anti‐amyloid beta‐proteins (Aβ) monoclonal antibodies for AD patients have been developed. Among the immunotherapeutic agents, lecanemab is an anti‐Aβ monoclonal antibody that binds to Aβ protofibrils (Aβ PFs), which is an intermediate molecule in Aβ species. Although Aβ PFs have been regarded as pathogenic aggregates among the Aβ species, the precise mechanisms of the molecular functions of Aβ PFs in the Aβ polymerization process have not been clarified. This research aims to reveal the molecular interactions of Aβ PFs against Aβ aggregation cascades in the mixed samples of low molecular weight (LMW) Aβ42 and different concentrations Aβ42 mature fibril (MF) seeds.

**Method:**

After confirming the morphological characteristics of Aβ PFs, we performed thioflavin T (ThT) assays using mixed samples containing LMW Aβ42 and MF seeds in various conditions in both the absence and the presence of Aβ PFs. The structural conversions of Aβ42 aggregates were evaluated using circular dichroism (CD) spectroscopy. Continuous observations of the fibril formations in the different Aβ samples were performed using high‐speed atomic force microscopy (HS‐AFM). Statistical analysis between two data was performed using a Mann‐Whitney *U*‐test. Statistical analysis among multiple data was performed using one‐way factorial ANOVA followed by Tukey post hoc comparisons.

**Result:**

We could confirm Aβ PFs with clavate‐shaped structures. The results of the ThT assays and CD spectroscopy showed that the presence of 2% Aβ PFs significantly accelerated Aβ aggregations with rapid conformational changes in the LMW Aβ42 sample. Aβ PFs did not promote Aβ aggregations in the mixed sample of LMW Aβ42 in the presence of 10% MF seeds, and inhibited the fibril elongations in the mixed sample of LMW Aβ42 in the presence of 2% MF seeds. In HS‐AFM images, the interactions of LMW Aβ42 and attached Aβ PFs on mica showed hyperbolic fibril formations.

**Conclusion:**

Aβ PFs have potencies to accelerate LMW Aβ42 aggregation, and there would be possible mechanisms for Aβ PFs to promote LMW Aβ42 aggregations resulting from the superficial interactions between and Aβ PFs and LMW Aβ42.